# Supplementary material for: A hybrid and poly-polish workflow for the complete and accurate assembly of phage genomes: a case study of ten przondoviruses
Source: Microb Genom. 2023 Jul 18;9(7):mgen001065. doi: 10.1099/mgen.0.001065 (PMC10438801; doi:10.1099/mgen.0.001065)
Supplement: Supplementary material 1 [file mgen-9-1065-s001.pdf]

SUPPLEMENTAL INFORMATION

Supplemental tables

Table S1. Metadata for bacterial strain collection

Bacterial strains	Code or Description	Source	K locus type	MLST	Accession	Reference
Clinical isolates						
K. aerogenes	CL1	Child	-	-	-	-
K. aerogenes	CL2	Child	-	-	-	-
K. aerogenes	CL3	Child	-	-	-	-
K. grimontii	P038I	Infant faeces	None	None	QFVM00000000	(25)
K. grimontii	P043G P	Infant faeces	KL169	ST216	QFVL00000000	(25)
K. michiganensis	M7 21 2 #35	Infant faeces	None	ST85	-	-
K. michiganensis	P049A W	Infant faeces	KL102	None	QFUG00000000	(25)
K. michiganensis	P095L Y	Infant faeces	KL109	ST157	QFTT00000000	(25)
K. oxytoca	M59 22 8 KoN	Infant faeces	KL74	ST215	-	-
K. oxytoca	M59 22 8 KoA	Derived from KoN	KL74	ST215	-	-
K. oxytoca	CL4	Child	-	-	-	-
K. oxytoca	CL5	Child	-	-	-	-
K. pneumoniae	CL6	Child	-	-	-	-
K. pneumoniae	CL7	Child	-	-	-	-
K. pneumoniae	M26 18 1	Infant faeces	None	ST432 (CG1777)	-	-
K. pneumoniae	M26 18 2 #21 KpnN	Derived from M26 18 1	None	ST432 (CG1777)	-	-
K. pneumoniae	M26 18 2 #21 KpnA	Derived from KpnN	None	ST432 (CG1777)	-	-
K. pneumoniae	P008E 1	Infant faeces	KL10	ST461	QFUX00000000	(25)
K. quasipneumoniae	P057K W	Infant faeces	KL11	ST4326-2LV	QFVB00000000	(25)

K. variicola	CL8	Child	-	-	-	-
Food isolates*						
K. pneumoniae	133-137-25	Chicken meat product	KL8	ST453	SAMN33593347	-
K. pneumoniae	148-152-26	Chicken meat product	KL21	ST290	SAMN33593348	-
K. pneumoniae	0311-342-6	Pork meat product	KL24	ST15 (CG15)	SAMN33593349	-
K. pneumoniae	H-26-2393-2397-33	Pork meat product	None	ST15 (CG15)	SAMN33593350	-
K. pneumoniae	H-27-MC778-782col38B	Chicken meat product	KL16	ST14 (CG15)	SAMN33593351	-
Misc. isolates						
K. pneumoniae	ST38 01	Unknown	None	ST134 (CG1)	-	-
Type isolates						
K. aerogenes	DSM 30053	DSMZ	None	ST134 (CG1)	CP002824	(26)
K. pneumoniae	DSM 30104	DSMZ	KL3	ST3 (CG3)	AJJI01000001	(27)
K. pneumoniae	NC 13438	NCTC	KL107	ST258 (CG258)	UGKS01000005	(28)
K. variicola	DSM 15968	DSMZ	KL16	ST2263	CP010523	(29)
Wastewater isolates						
K. huaxiensis	0220.RS02	Raw sewage	None	None	-	This study
K. michiganensis	0220.ML02	Mixed liquor	None	None	-	This study
K. michiganensis	0220.ML04	Mixed liquor	None	None	-	This study
K. pneumoniae	0220.ML03	Mixed liquor	KL38	ST37 (CG37)	-	This study
K. pneumoniae	0220.RS01	Raw Sewage	KL30	ST1618	-	This study
K. ornithinolytica	0220.AF01	Aeration feed	None	None	-	This study
K. ornithinolytica	0220.ML01	Mixed liquor	KL144	None	-	This study

*, food isolates overall BioProject accession PRJNA941224; MLST, multilocus sequence type; ST, sequence type; CG, clonal group; -, no data.

Table S2. HYPPA assembly details for all ten przondoviruses in this study

Phage lab code	Phage name	Assembler	No. contigs	No. of reads	Read length N50	Longest read (bp)	Long-read coverage	Size pre-polishing (bp)	Size post-polishing* (bp)	Final assembly (bp)	DTR size (bp)
CE3	Oda	Canu	1	1,825	12,634	41,869	146x	41,761	41,769	41,642	181
CE8	Toyotomi	Flye	1	365,482	39,443	106,111	117,981x	41,249	41,286	41,268	180
CE9	Mera	Flye	3[a]	13,252	12,497	58,219	8x	41,454	40,619	41,400	180
CE10	Speegle	Flye	1	47,260	12,610	81,712	23x	41,394	41,395	41,395	180
CE11	Cornelius	Canu	1	5,719	1,388	47,884	118x	40,555	40,564	40,437	180
CE12	Tokugawa	Canu	1	1,018	19,093	41,581	92x	41,532	41,542	41,414	181
CE15	Saitama	Canu	1	19,217	2,597	43,752	661x	46,600	46,613	40,741	181
CE20	Emom	Canu	1	2,683	4,862	41,521	52x	40,903	40,915	40,788	183
CE22	Amrap	Flye	2[a]	30,181	717	40,963	27x	41,328	41,331	41,209	182
CE30	Whistle	Canu	1	9,239	1,614	46,197	200x	40,855	40,862	40,735	181

*, Post-polishing was 2 x long-read and 2 x short-read polishing, as per the HYPPA workflow; [a], genome contained within the first contig.

Table S3. Type and number of assembly errors found in short-read-only and long-read only assemblies combined, prior to application of HYPPA

Phage lab code	Phage name	Assembler	No. repeat region errors	No. homo-polymer errors	No. SNP errors	No. indels[a]	Of which in long-read-only	Of which in short-read-only
CE3	Oda	Canu	2	6	3	1	9	3
CE8[b]	Toyotomi	Flye	2	28	35	1	66	N/A
CE9	Mera	Flye	4	3	2	1	4	6
CE10	Speegle	Flye	2	1	2	1	3	3
CE11	Cornelius	Canu	4	8	1	0	9	4
CE12	Tokugawa	Canu	4	9	0	0	9	4
CE15	Saitama	Canu	4	8	0	0	8	4
CE20	Emom	Canu	4	9	1	0	10	4
CE22	Amrap	Flye	2	2	1	1	3	3
CE30	Whistle	Canu	4	7	0	0	7	4

[a], indels included insertions/deletions separate to both repeat region errors and SNP errors (≥2 bp long). [b], no short-read-only assembly for CE8, errors based only on long-read-only assembly prior to HYPPA.

Table S4. Alternative long-read-only assembly details for all ten przondoviruses in this study

Phage lab code	Phage name	Assembler	No. contigs	Size (bp)	Size post-polishing (bp)
CE3	Oda	Flye	1	41,116	41,119
CE8	Toyotomi	N/A	N/A	N/A	N/A
CE9	Mera	Canu	1	41,527	40,237
CE10	Speegle	Canu	5	31,413[a]	N/A
CE11	Cornelius	Flye	1	40,487	N/A
CE12	Tokugawa	Flye	1	40,791	40,796
CE15	Saitama	Flye	6	40,551[b]	40,558
CE20	Emom	Flye	1	41,250	N/A
CE22	Amrap	Canu	1	25,100[a]	N/A
CE30	Whistle	Flye	1	40,843	N/A

N/A, not performed; [a], incomplete assembly; [b], genome contained within the first contig.

Table S5. Short-read-only assembly details for all ten przondoviruses in this study

Phage lab code	Phage name	Assembler	No. contigs	No. reads	Short-read coverage	Size (bp)	Size post-curation (bp)
CE3	Oda	Shovill	1	527,773	x3737	41,540	41,641
CE8	Toyotomi	Shovill	46	396,472	x2832	*	N/A
CE9	Mera	Shovill	1	102,411	x737	41,315	N/A
CE10	Speegle	SPAdes	56	55,213	x401	41,270	N/A
CE11	Cornelius	Shovill	1	353,905	x2608	40,336	N/A
CE12	Tokugawa	Shovill	1	414,141	x2935	41,312	41,413
CE15	Saitama	Shovill	4	1,181,498	x256	40,633	N/A
CE20	Emom	Shovill	1	1,027,070	x5627	40,692	N/A
CE22	Amrap	Shovill	1	902,433	x1846	41,106	N/A
CE30	Whistle	Shovill	1	1,914,368	x13,511	40,641	N/A

N/A, not performed; *, no single contig contained the entire genome for CE8: assembly was performed with subsampled reads in multiple iterations, but this did not improve the assembly quality.

Table S6. Hybrid assembly details for all ten przondoviruses in this study

Phage lab code	Phage name	Assembler	No. contigs	Size (bp)
CE3	Oda	N/A	N/A	N/A
CE8	Toyotomi	SPAdes	64	40,626[a]
		Unicycler	6	40,563[a]
CE9	Mera	SPAdes	124	41,329[b]
CE10	Speegle	SPAdes	74	41,270[c]
CE11	Cornelius	N/A	N/A	N/A
CE12	Tokugawa	N/A	N/A	N/A
CE15	Saitama	N/A	N/A	N/A
CE20	Emom	N/A	N/A	N/A
CE22	Amrap	N/A	N/A	N/A
CE30	Whistle	N/A	N/A	N/A

N/A, not performed; [a], genome contained within the first two and largest contigs; [b], genome contained within the first seven and largest contigs; [c], genome contained with the first contig.

Table S7. Metadata for a selection of publicly-available *Autographiviridae* phages downloaded from NCBI for comparative genomics

Phage name	Accession	Genome size (bp)	DTR (bp)[a]	Reference
Studiervirinae, *Przondovirus*				
Escherichia phage K30	HM480846	40,940	Yes (393)	(67)
Klebsiella phage 2044-307w	MF285615	40,048	Yes (181)	-
Klebsiella phage Henu1	MK203841	40,352	No	(68)
Klebsiella phage K5	KR149291	41,698	Yes (392)	-
Klebsiella phage K11	EU734173	41,181	Yes (180)	(69)
Klebsiella phage KP32	GQ413937	41,119	No	(20)
Klebsiella phage KPN3[b]	MN101227	38,503	No	-
Klebsiella phage Kund-ULIP47	MK380015	41,397	Yes (180)	(70)
Klebsiella phage Kp_Pokalde_002	MT425185	41,816	Yes (180)	
Klebsiella phage SH-KP152226	MK903728	41,420	Yes (180)	(71)
Klebsiella phage vB_KpnP_PRA33	KY652723	40,605	No	(72)
Klebsiella phage vB_KpnP_BIS33	KY652725	41,697	No	(72)
Klebsiella phage vB_KpnP_KpV767	KX712070	40,395	Yes (180)	-
Klebsiella phage KMI1[b]	MN052874	37,414	No	-
Klebsiella phage SH-Kp 152410	MG835568	40,945	No	-
Klebsiella phage K5-2	KY389315	41,116	Yes (177)	(66)
Klebsiella phage K5-4	KY389316	40,163	Yes (179)	(66)
Klebsiella phage vB_Kpn-VAC71	MZ571832	40,388	No	-
Klebsiella phage vB_KpnP_IME205	KU183006	41,310	No	(73)
Klebsiella phage IME264	OL799328	40,671	No	-
Studiervirinae, *Teetrevirus*				
Salmonella phage phiSG-JL2	EU547803	38,815	Yes (230)	(74)
Citrobacter phage SH1	KU687347	39,434	Yes (231)	(75)
Klebsiella phage vB_KpnP-VAC1	MZ428229	39,371	No	(76)
Studiervirinae, *Teseptimavirus*				
Escherichia phage T7	V01146	39,937	Yes (160)	(77)
Enterobacteria phage 13a	EU734174	38,841	Yes (170)	-
Studiervirinae, *Apidecimavirus*				
Yersinia phage vB_YenP_AP10	KT852574	39,235	Yes (193)	-
Studiervirinae, *Berlinvirus*				
Yersinia phage Berlin	AM183667	38,564	Yes (226)	-
Slopekvirinae, *Drulisvirus*				
Klebsiella phage F19	KF765493	43,766	Yes (119)[c]	-
Molineuxvirinae, *Zindervirus*				
Salmonella phage SP6	AY370673	43,769	Yes (174)	(78)

[a], Yes, complete genomes were those with the DTRs present that may or may not be annotated; No, genomes without DTRs present and therefore deemed incomplete. [b], Klebsiella phages KPN3 and KMNI1 were deemed incomplete due to potential errors in multiple genes. [c], DTRs were annotated but only the first 94 bp of the 119 bp annotation are homologous. -, no data.

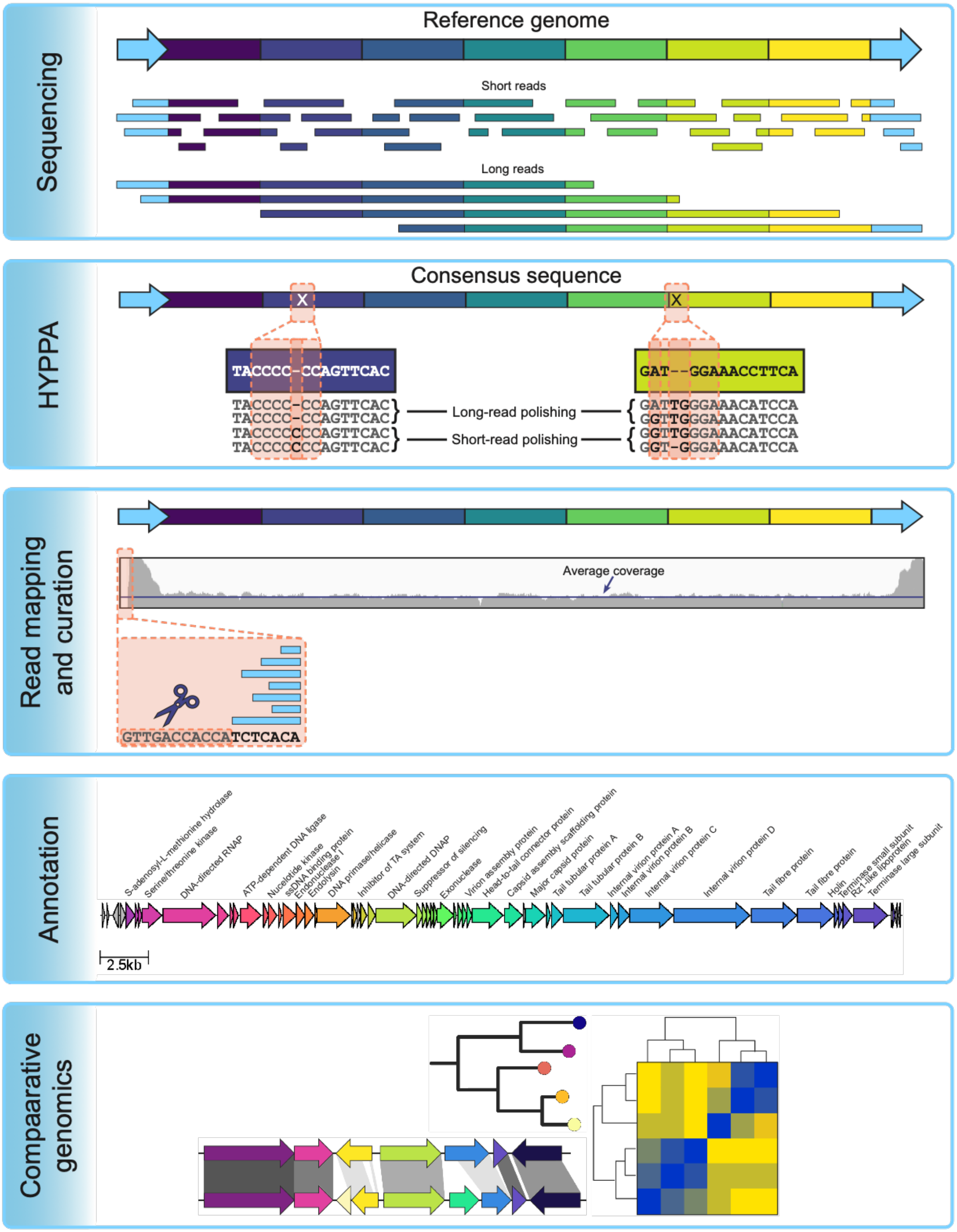

Fig. S1. Workflow for phage genome assembly and analysis. Both long-read and short-read sequencing are recommended, followed by our HYPPA workflow for high quality phage genome assembly. Read mapping for short-read data and manual curation can correct any errors that were missed during polishing, followed by annotation and then comparative genomics.

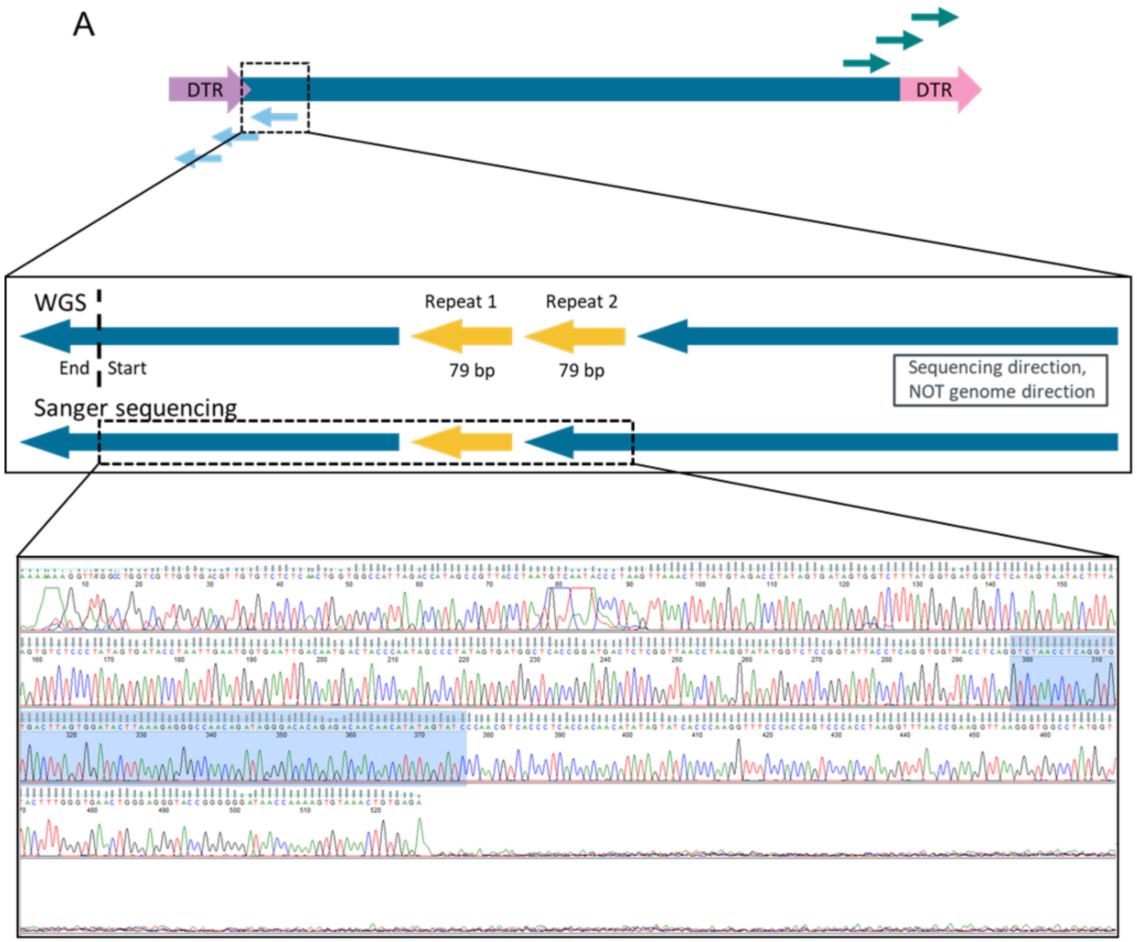

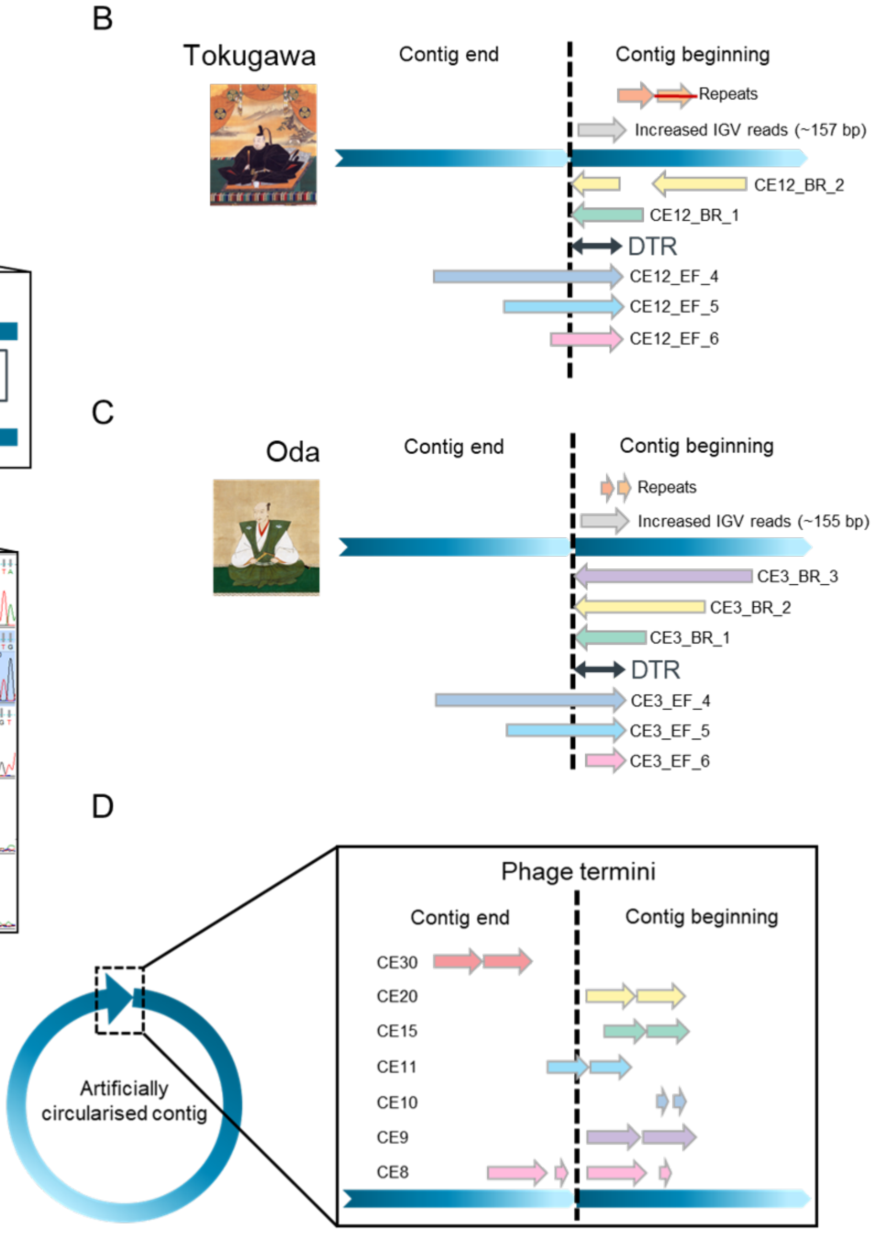

Fig. S2. Schematic of primer walking and Sanger sequencing validation of the HYPPA workflow. (A) Three primers were designed to "walk" the phage termini (top panel). Results for *Klebsiella* phage Tokugawa showed that there were two 79 bp repeats in the short read - only assembly data (middle panel), where only one was present in the Sanger sequencing data (middle and bottom panel), as is highlighted in the chromatograph, suggesting an assembly error. **(B)** Schematic of the primer walking validation for *Klebsiella* phage Tokugawa showing where the 79 bp repeats were, and that one was introduced in error. The two reverse and three forward primers marked the area where the DTR began and ended, and corresponded to the area of increased IGV reads. **(C)** Schematic of the primer walking validation for *Klebsiella* phage Oda using three primers for each terminus. The assembly had small 13 bp repeats that were confirmed as genuine following Sanger sequencing. **(D)** Schematic of the phage termini with all the larger repeats introduced in error during short-read-only assembly. The small repeats found in CE8 and CE10 were genuine small repeats of 13 bp.

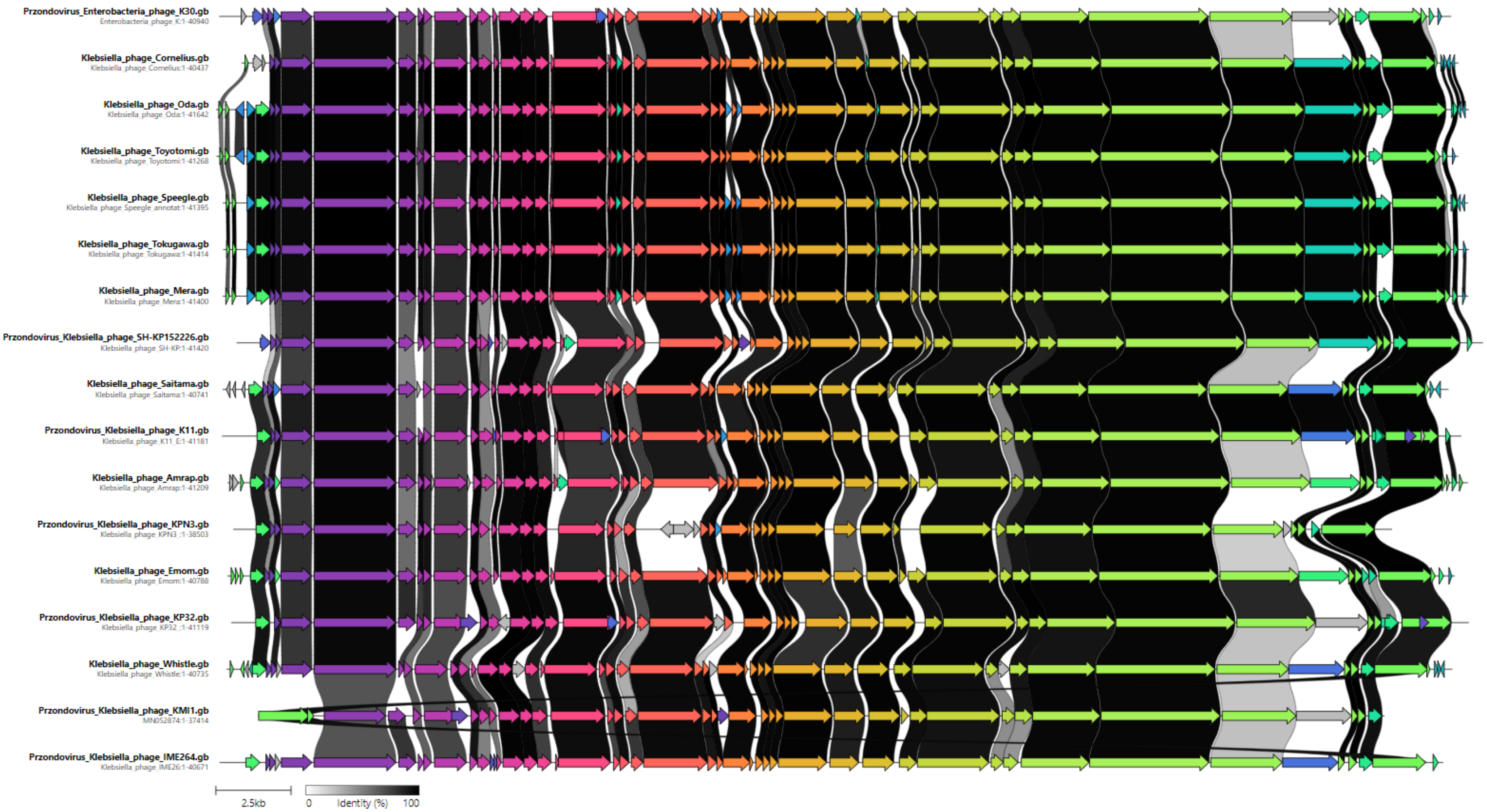

Fig. S3. Genome map and gene clustering for przondoviruses in the collection and a selection of related phages including relatives with potential errors. Arrows represent coding sequences and pairwise comparisons of gene similarities are indicated by percentage identity given as links in greyscale, with darker shading representing areas of higher similarity. Genes without any sequence similarity are indicated without links. DTRs are not annotated. Potential assembly/sequencing errors in reference phages: i) several missing genes including S-adenosyl-L-methionine hydrolase in phage SH-KP152226; ii) incomplete annotation of the DNA polymerase and missing tail tubular protein A in phage KPN3; iii) incorrect start site of phage KMI1, along with incomplete DNA polymerase and several missing genes, including S-adenosyl-L-methionine hydrolase and serine/threonine kinase.

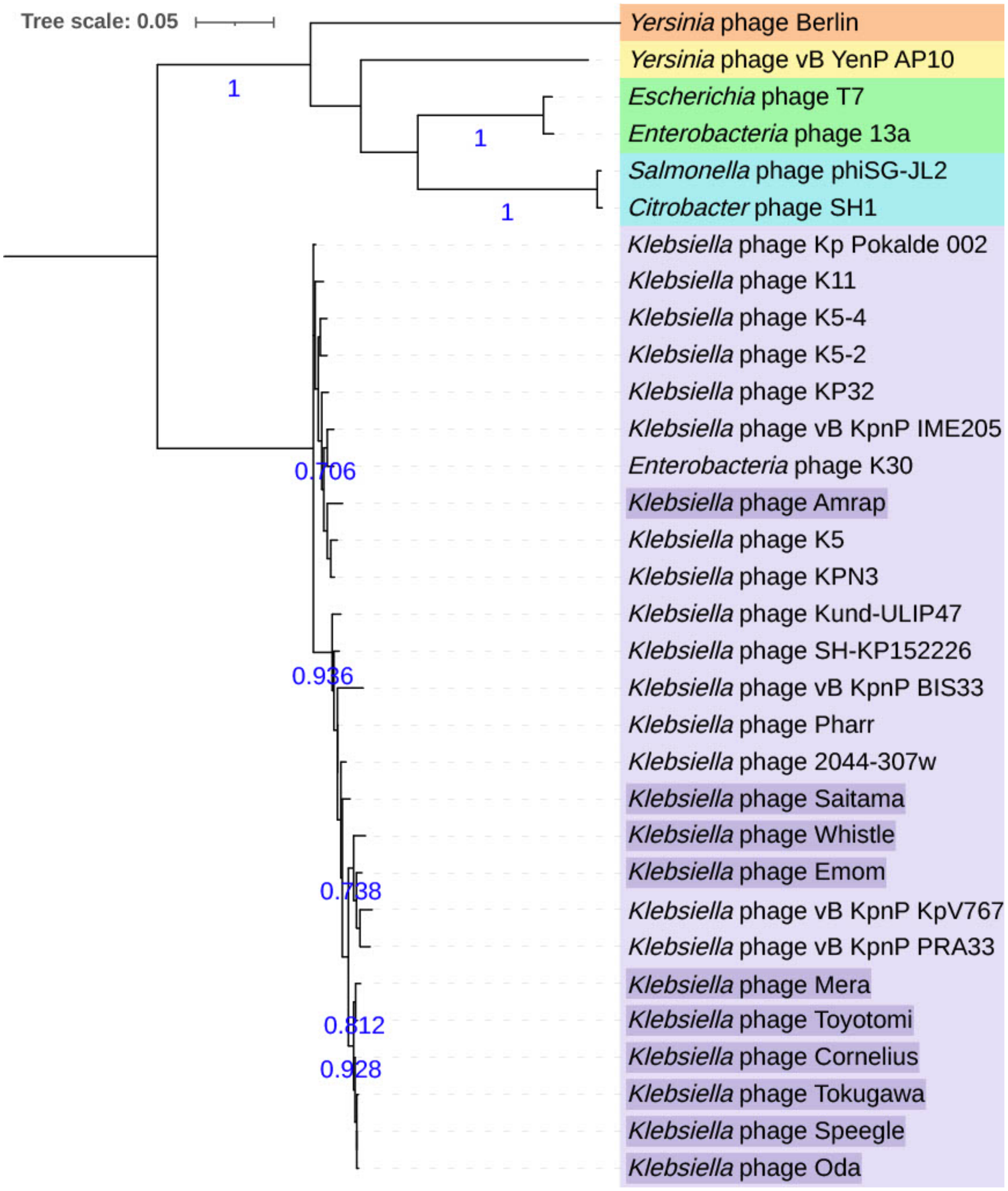

Fig. S4. Maximum-likelihood phylogeny of the RNAP for przondoviruses in this study and a selection of related *Studiervirinae* phages. All phages from this study (purple highlight) clustered with related przondoviruses (purple). Outgroups, *Berlinvirus* (orange), *Apidecimavirus* (yellow), *Teseptimavirus* (green), and *Teetrevirus* (blue). Tree is midpoint rooted. Bootstrap support values at ≥0.7 are given in blue (500 replicates). Scale bar represents number of amino acid substitutions per site.
